# Adherence to Self-Monitoring via Interactive Voice Response Technology in an eHealth Intervention Targeting Weight Gain Prevention Among Black Women: Randomized Controlled Trial

**DOI:** 10.2196/jmir.2996

**Published:** 2014-04-29

**Authors:** Dori M Steinberg, Erica L Levine, Ilana Lane, Sandy Askew, Perry B Foley, Elaine Puleo, Gary G Bennett

**Affiliations:** ^1^Duke Obesity Prevention ProgramDuke Global Health InstituteDuke UniversityDurham, NCUnited States; ^2^Department of Psychology and NeuroscienceDuke UniversityDurham, NCUnited States; ^3^School of Public Health and Health SciencesDepartment of BiostatisticsUniversity of Massachusetts AmherstAmherst, MAUnited States

**Keywords:** eHealth, obesity, interactive voice response, self-monitoring

## Abstract

**Background:**

eHealth interventions are effective for weight control and have the potential for broad reach. Little is known about the use of interactive voice response (IVR) technology for self-monitoring in weight control interventions, particularly among populations disproportionately affected by obesity.

**Objective:**

This analysis sought to examine patterns and predictors of IVR self-monitoring adherence and the association between adherence and weight change among low-income black women enrolled in a weight gain prevention intervention.

**Methods:**

The Shape Program was a randomized controlled trial comparing a 12-month eHealth behavioral weight gain prevention intervention to usual care among overweight and obese black women in the primary care setting. Intervention participants (n=91) used IVR technology to self-monitor behavior change goals (eg, no sugary drinks, 10,000 steps per day) via weekly IVR calls. Weight data were collected in clinic at baseline, 6, and 12 months. Self-monitoring data was stored in a study database and adherence was operationalized as the percent of weeks with a successful IVR call.

**Results:**

Over 12 months, the average IVR completion rate was 71.6% (SD 28.1) and 52% (47/91) had an IVR completion rate ≥80%. At 12 months, IVR call completion was significantly correlated with weight loss (*r* =−.22; *P*=.04) and participants with an IVR completion rate ≥80% had significantly greater weight loss compared to those with an IVR completion rate <80% (−1.97 kg, SE 0.67 vs 0.48 kg, SE 0.69; *P*=.01). Similar outcomes were found for change in body mass index (BMI; mean difference −0.94 kg, 95% CI −1.64 to −0.24; *P*=.009). Older, more educated participants were more likely to achieve high IVR call completion. Participants reported positive attitudes toward IVR self-monitoring.

**Conclusions:**

Adherence to IVR self-monitoring was high among socioeconomically disadvantaged black women enrolled in a weight gain prevention intervention. Higher adherence to IVR self-monitoring was also associated with greater weight change. IVR is an effective and useful tool to promote self-monitoring and has the potential for widespread use and long-term sustainability.

**Trial Registration:**

Clinicaltrials.gov NCT00938535; http://www.clinicaltrials.gov/ct2/show/NCT00938535 (Archived by WebCite at http://www.webcitation.org/6P1FFNJRs).

## Introduction

During the past decade, a growing body of evidence has demonstrated the efficacy of electronic health (eHealth) interventions for weight management [1,2]. These interventions have been tested on a range of digital platforms (eg, Web, text messaging, mobile applications) and show promise in overcoming some of the challenges inherent with traditional weight loss interventions (eg, cost, reach, scale, expertise) [3]. Although intervention designs vary considerably, evidence suggests that eHealth interventions can produce clinically meaningful weight loss outcomes [2]. Such findings are promising, particularly among racial/ethnic minority groups who have high utilization of mobile and Web-based technologies [4,5] and bear the most burden from obesity [6].

Most weight control interventions promote some form of self-monitoring, usually recommending that participants provide detailed reports of diet, physical activity, weight, or obesity-related risk behaviors (eg, sugar sweetened beverages) using paper diaries. Indeed, evidence indicates that self-monitoring is highly predictive of weight loss success [7,8]. Despite its effectiveness, adherence to traditional paper-based approaches declines over time [7,9].

eHealth approaches offer unique features that may help abate the usual decline in self-monitoring adherence. eHealth self-monitoring strategies (eg, Web-based dietary monitoring, mobile applications with food diaries, activity trackers) are often more portable, allow for more proximal reporting, can prompt individuals based on timing, context, or participant progress, and have the ability to provide immediate and tailored feedback [10]. Furthermore, qualitative evidence indicates that participants are more receptive to eHealth approaches compared to paper-based methods [11,12]. Identifying effective eHealth self-monitoring strategies that can further enhance adherence is important because evidence consistently demonstrates that the magnitude of behavior change in eHealth interventions is largely dependent on the level of participant adherence or engagement with the intervention [1].

Interactive voice response (IVR) is one such eHealth self-monitoring approach. IVR allows participants to interact with a computer system via outbound or inbound telephone calls using the keypad or speech. Use of IVR is ubiquitous in the wider consumer market (eg, used with telephone banking, checking airline flight status, automated appointment reminders with health systems, etc) and, given its widespread familiarity, might be an effective way to collect self-monitoring data within health interventions.

IVR has been used in a variety of clinical contexts as a means of both delivering intervention content and collecting data [13-17]. IVR may have a number of distinct advantages over the use of other eHealth modalities [18]. It can be particularly useful for low literacy populations [19]: the task of listening to a voice prompt and responding with a simple numerical answer may be far less cognitively and numerically demanding than producing detailed reports of self-monitoring data (eg, caloric intake or fat intake). IVR calls may also be less time-consuming than other modalities for self-monitoring that might require participants to keep a paper food diary, log on to an online system, or conduct an extensive search for required numerical data (eg, calorie intake) [20,21]. Additionally, IVR systems can be used to provide dynamic and immediate feedback in response to self-monitoring data. Indeed, a review of the evidence suggests improvements in health outcomes with the use of IVR for self-monitoring [17].

Despite the growing literature surrounding the use of IVR technologies, limited evidence exists on the use and effectiveness of IVR for weight control [22,23]. We sought to examine the association between IVR self-monitoring and weight change among socioeconomically disadvantaged black women enrolled in the Shape Program (“Shape”). Shape was an 18-month randomized controlled trial comparing an eHealth weight gain prevention intervention to usual care among overweight and class 1 obese black female primary care patients [24]. Intervention participants self-monitored their behavior change goals via weekly IVR phone calls. Findings from Shape indicate that the intervention was successful in preventing weight gain among intervention participants relative to those receiving standard primary care [25]. At 12 months, a larger proportion of intervention participants (62%) were at or below their baseline weight, compared to those in usual care (45%; *P*=.02). Similar findings were observed at 18 months [25]. In the present analysis, we describe patterns of IVR self-monitoring adherence over time, examine relevant predictors of adherence, and explore the association between adherence and weight change.

## Methods

### Study Design

**Figure 1:**
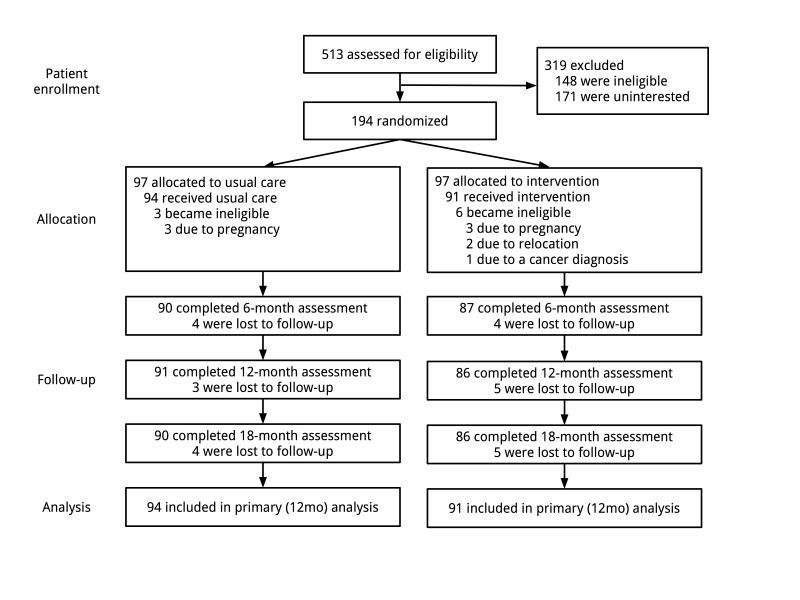
Participant enrollment and retention (CONSORT).

The Shape Program design and methods have been detailed elsewhere [24,25]. Starting in December 2009, participants were recruited via mail from five community health centers operated by Piedmont Health (PHS) in central North Carolina. Participants were black women, aged 25 to 44 years, with a body mass index (BMI) of 25-34.9 kg/m^2^. Following eligibility screening, informed consent, and baseline measures, we randomized participants (n=194) to either the Shape intervention or usual care arm. All participants were re-evaluated at 6 and 12 months, with additional follow-up at 18 months post randomization (Figure 1). Final assessments were completed in October 2012. The relevant university and health system review boards approved all study procedures.

### Intervention Description

The Shape intervention included five main components: (1) behavior change goals known to promote weight change, (2) self-monitoring of these goals via weekly IVR phone calls, (3) tailored skills training materials, (4) monthly interpersonal counseling calls with a PHS registered dietitian (“Shape coach”), and (5) a 12-month YMCA membership. Usual care group participants received routine standard of care from their providers at PHS.

The intervention utilized the interactive obesity treatment approach (iOTA), which has been extensively tested in previous studies [23,26]. iOTA uses an algorithm to prescribe tailored, evidence-based behavior change goals in order to create a sufficient caloric deficit to produce weight change (eg, five or more fruits and vegetables/day, no fast food, no sugar sweetened beverages, walking 7000 steps/day). Participants do not self-select which goals to track. Rather, participants are assigned through the algorithm three behavior change goals from a library of 21 goals based on their self-efficacy and readiness, and the potential for the goal to produce a caloric deficit. For novelty and to ensure that participants changed multiple behaviors, goals were updated every two months based on the output from the original algorithm.

**Figure 2:**
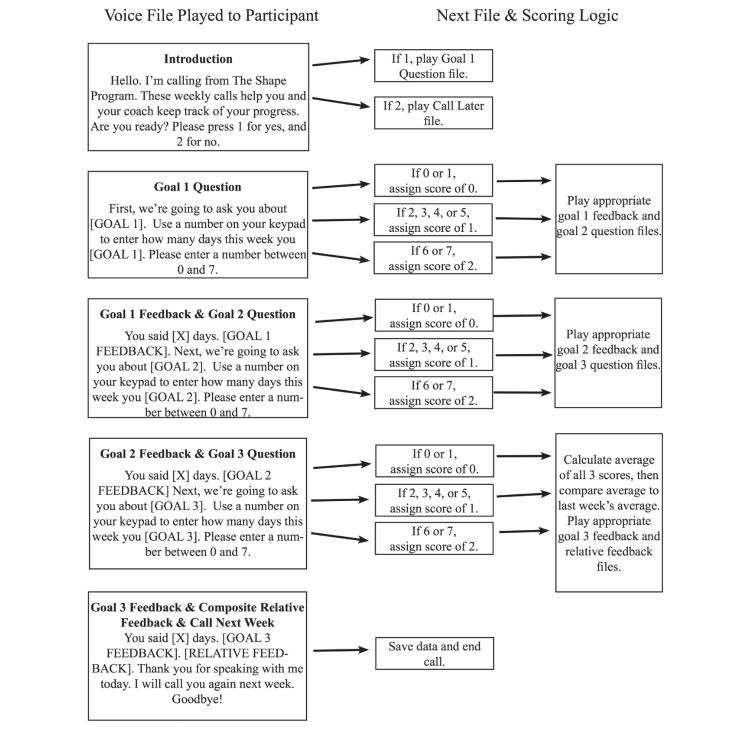
IVR call logic.

Participants self-monitored these goals throughout the 12-month intervention via weekly IVR phone calls. The IVR calls were on average 2-4 minutes in duration. The IVR system called each participant once a week at a predetermined time. If a participant was not reached on the initial attempt, an extensive retry protocol was put into place, with a maximum of 16 attempts over two days. As shown in Figure 2, once the IVR system made contact with the participant, it asked how many days this past week they achieved each of their assigned behavioral goals (eg, “this past week, how many days did you drink sugary drinks?”). When relaying the self-monitoring data, participants had the option to reference a paper-based goal-tracking sheet that included daily reporting of these goals. For example, if a participant’s goal was “no sugary drinks”, she might report in her paper log each day whether or not she drank any sugary drinks. At the end of the week, she summed the days she drank any sugary drinks. The IVR questions were phrased in such a way as to be concrete and dichotomous, making it easier to remember goal achievement during the weekly calls (eg, “How many days this week did you drink sugary drinks?”). Thus, the paper log was considered optional. Participants were encouraged to use the paper logs only as a means to help them relay the data through the IVR system. However, the IVR system was designed to be simple enough to use without the paper logs

After self-monitoring data was collected and stored in a study database, brief tailored feedback and short skills training tips were immediately provided. Based on goal performance, a score was assigned to each goal (eg, if a participant reported drinking sugary drinks zero days last week, she received a high score of 2). An average goal attainment score across all behaviors determined feedback messages, which were pulled from a pre-determined set of feedback messages that were transferred to voice files (eg, “Looking at all of your goals together,…”)*.* The algorithm included a component to prevent repeated feedback messages within a set number of weeks.

Feedback messages (Figure 2) included a relative comparison to the previous week’s self-monitoring data (eg, “...you did better than last time—great job!” or “...you’re not doing quite as well as you did last week. Let’s turn this slip around”) and specific tips on how to boost performance (eg, “Think about the things that you did on the day you met your goal and how you can you do those things more often next week” or “You’re doing great! Stick with it and you can get to 7 days next week”). A sample call can be heard in Multimedia Appendix 1. Messaging content was selected from a large library of feedback messages from previous studies using the iOTA approach in similar populations [23,26,27]. All IVR call logic was rigorously tested and continuous quality control protocols were performed to ensure fidelity to protocol. Adherence to IVR tracking was also encouraged during the monthly coaching calls and participants were aware that IVR data were relayed to the coaches. The coaches had access to IVR completion rates for each participant and provided feedback and counseling on strategies to maintain weekly tracking.

### Measures

#### Self-Monitoring

The IVR system collected and stored response data to each of the three goals, as well as data on call time and call length in a study database. Self-monitoring adherence was calculated as the proportion of intervention participants who successfully completed IVR calls over the number of expected to complete a call by study week. Calls were deemed successful once data on each of the three goals were received. Some participants (n=9) requested to suspend or stop intervention activities and/or experienced technical problems with the IVR system. We will assess self-monitoring adherence with and without these participants. Although IVR was the primary self-monitoring mode, participants were given the option to use paper logs daily. At 12 months, participants self-reported via an online questionnaire the average number of days per week they used the paper log (ie, 5-7 days per week, 3-4 days per week, 1-2 days per week, or not at all). We also assessed perceptions about IVR self-monitoring at 12 months using an online questionnaire. Participants reported agreement via a 6-point Likert scale that ranged from “strongly agree” to “strongly disagree” on various statements assessing perceptions about IVR self-monitoring (eg, the tracking calls made it easy for me to keep track of my behaviors, the tracking calls were difficult to use, or I enjoyed receiving the tracking calls).

#### Determinants of Adherence

We selected several relevant baseline sociodemographic variables and psychosocial constructs that might predict self-monitoring adherence based on behavior change theories [28] and previous literature suggesting psychosocial variables such as stress [29], perceived social support [30], and self-efficacy [31] may impact engagement and outcomes in weight control trials. Demographic variables such as age, marital status, educational attainment (five categories*:* less than high school, high school, vocational school, some college, or college degree or more), employment, and income (US $30,000/year or more vs less than US $30,000/year) were collected. The 8-item Patient Health Questionnaire assessed the presence of depressive symptoms [32]. The 19-item subscale from the Medical Outcomes Study (MOS-SSS) was utilized to assess availability of social support. Four subscales were included: emotional/informational, tangible, affectionate, and positive social interaction [33]. A 16-item questionnaire that was used in the CARDIA study was administered to measure frequency of stressful life events [34]. The Marcus self-efficacy for exercise questionnaire was used to assess confidence in one’s ability to exercise when tired, in a bad mood, don’t have time, on vacation, or when it is raining/snowing [35]. All measures were administered via online questionnaires.

Given that the Shape coaches were able to view IVR call patterns, coaching call completion was assessed as another potential predictor. The monthly coaching calls were delivered via a similar software system as the IVR calls. As a result, we were able to capture start and end time of each call. We used call duration data, along with coach documentation of topics covered, as a proxy of completion. Coaching call completion was operationalized as the actual number completed over the number expected.

#### Anthropometrics

Study staff collected weight and height data at baseline, 6, and 12 months within study offices. Body weights were measured to the nearest 0.1 kg using a portable electronic scale (Seca Model 876) and heights were measured using a calibrated wall-mounted stadiometer (Seca 214) [36].

#### Statistical Analyses

All analyses were conducted within the intervention group only (n=91). Descriptive statistics were conducted to characterize the sample and examine average IVR completion rate over the 12-month period. IVR adherence was dichotomized using a median split (80% or more) to examine differences in outcomes among high completers compared to those below the median. Adherence was also analyzed as tertiles of successful weekly IVR calls. We conducted bivariate analyses using *t* tests and chi-square to examine potential predictors of average IVR completion rate and categories of IVR completion. Pearson correlations examined the relationship between weight and BMI change and IVR call completion rate. ANOVA (analysis of variance) analyzed differences in weight change and BMI change among high and low IVR completers, tertiles of IVR completion, and categories of IVR and paper log completion. Last, given that participants were nested within health centers, we tested the intraclass correlation (ICC) and did not find a meaningful effect of the nested design (ICC=.07; 95% CI 0.01-0.38). Therefore, no further adjustment was required.

## Results

### Baseline Characteristics and Retention

Baseline characteristics and main outcomes have been reported in detail elsewhere [24,25]. Briefly, participants reported at baseline a mean age of 35.4 years (SD 5.5) and a mean BMI of 30.2 kg/m^2^ (SD 2.5). Most (71.4%, 130/182) were currently employed with an annual income <US $30,000/year (74.3%, 136/183). The majority (79.7%, 145/182) had less than a college degree. We retained 95.7% (177/185) of participants at 12- and 18-months post randomization (Figure 1) and there were no statistically significant differences in attrition between groups.

### Self-Monitoring Adherence

**Figure 3:**
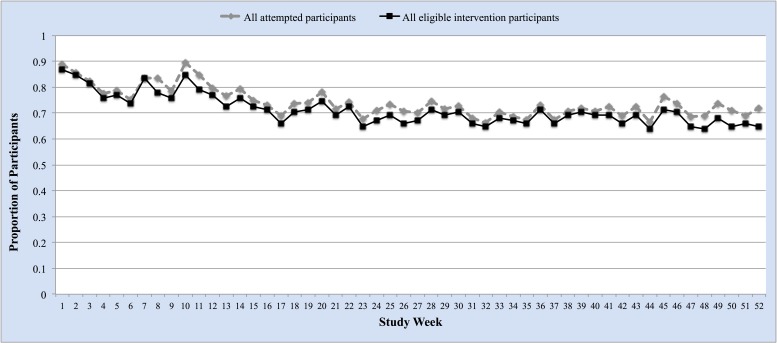
Proportion of participants who completed IVR calls by study week.

Figure 3 shows IVR adherence rates over time by study week. Among all intervention participants (n=91), the average IVR completion rate over 12 months was 71.6% (SD 28.1) with a weekly range from 52% to 96%. Similar results are seen among all attempted participants; this rate excludes participants at each study week that may have requested to suspend or stop intervention activities and/or experienced technical problems with the IVR system (82/91 at week 52). A total of 52% (47/91) of intervention participants had an IVR completion rate of 80% or more, and two-thirds (66%, 60/91) completed at least 60% of IVR calls. Throughout the 12-month period, 39% (20/52) of IVR calls were completed on the first attempt and 78% (40/52) of calls were completed by the third attempt. About half of participants (49%, 41/83) self-reported using the paper tracking log at least 5 days each week in order to relay behavioral goal attainment to the weekly IVR calls.

### Predictors of Adherence

Age and education were the only sociodemographic or psychosocial variables that significantly predicted IVR adherence. High IVR completers (≥80%) were older (*P*=.03) and more educated (*P*=.003) than participants who completed <80% of IVR calls. Similar findings were found when assessing IVR call completion as a continuous variable (data not shown). Intervention participants completed 82% (10/12) of counseling calls during the 12-month intervention period. Coaching call completion was highly correlated with IVR completion rate (*r*=.77; *P*<.001). The percent of coaching calls completed was also predictive of the odds of completing 80% of IVR calls. Each 10% increase in coaching calls completed was associated with almost three times the odds of completing 80% or more of IVR calls (OR 2.57, 95% CI 1.59-4.26; *P*<.001).

### Self-Monitoring Adherence and Weight Change

IVR call completion was significantly correlated with 12-month weight loss (Spearman’s *r*=−.22; *P*=.04). Tables 1 and 2 show the associations between specific thresholds of IVR adherence and weight outcomes. At 12 months, participants with an IVR completion rate of at least 80% had greater weight loss outcomes compared to those with an IVR completion rate of less than 80% (mean difference −2.45 kg, 95% CI −4.37 to −0.54; *P*=.01). We observed similar findings for 12-month change in BMI (mean difference −0.94 kg/m^2^, 95% CI −1.64 to −0.24; *P*=.009). Although differences in weight change by tertiles of IVR call completion did not reach significance (Table 2), there were significant differences in BMI change between those who completed less than 60% of IVR calls and those who completed at least 93% of IVR calls (mean difference −0.87 kg/m^2^, 95% CI −1.73 to −0.01; *P*=.047). Similar results were seen when comparing those who completed less than 60% to 60-93% call completion (mean difference −0.88 kg/m^2^, 95% CI −1.76 to −0.002; *P*=.049).

As determined by self-reported paper log use at 12 months, the use of the paper logs did not enhance weight outcomes beyond what was achieved by the use of IVR. Although it did not reach statistical significance, at 12 months, participants with high IVR completion (≥80%) and high self-reported paper log use (≥ 5 days per week) lost 1.94 kg (SE 1.2), while those with lower IVR completion, but high self-reported paper log use gained 1.38 kg (SE 1.3) (mean difference −3.32 kg/m^2^, 95% CI −7.53 to 0.89; *P*=.17). Similar findings were seen when comparing participants with high IVR completion, but low self-reported paper log use [−2.04 kg (SE 0.76)] to those with lower IVR completion and low tracking log use [0.30 kg (SE 0.86); mean difference −2.34 kg/m^2^, 95% CI −6.35 to 1.67; *P*=.42].

**Table 1:** Change in weight and body mass index by IVR call completion (n=91).^a^

Anthropometric Changes	Time	IVR<80%, mean (SE) (n=44)	IVR ≥80%, mean (SE) (n=47)	Difference, mean (95% CI)^c^
**Change in weight, kg**				
	Month 6	−0.56 (0.57)	−1.16 (0.56)	−0.60 (−2.19 to 0.99)
	Month 12	0.48 (0.69)	−1.97 (0.67)	−2.45 (−4.37 to −0.54)
**Change in body mass index** ^b^				
	Month 6	−0.17 (0.21)	−0.34 (0.21)	−0.17 (−0.76 to 0.42)
	Month 12	0.25 (0.25)	−0.70 (0.25)	−0.94 (−1.64 to −0.24)

^a^Denominators vary because of missing data.

^b^Calculated as weight in kilograms divided by height in meters squared.

^c^Confidence intervals that do not contain zero have a *P* value <.05.

**Table 2:** Change in weight and body mass index by tertiles of IVR call completion (n=91).^a^

Anthropometric changes	Time				Difference between tertiles, mean (95% CI)^c^		
		Tertile 1 IVR<60% (n=30)	Tertile 2 IVR 60-92% (n=29)	Tertile 3 IVR ≥93% (n=32)	Between 1^st^ and 2^nd^	Between 1^st^ and 3^rd^	Between 2^nd^ and 3^rd^
**Change in weight, kg, mean (SE)**							
	Month 6	−0.46 (0.70)	−1.50 (0.71)	−0.69 (0.67)	−1.04 (−3.01 to 0.94)	−0.23 (−2.16 to 1.69)	0.81 (−1.14 to 2.75)
	Month 12	0.78 (0.85)	−1.60 (0.87)	−1.51 (0.82)	−2.39 (−4.80 to 0.02)	−2.29 (−4.64 to 0.06)	−0.09 (−2.28 to 2.46)
**Change in body mass index** ^b^ **, mean (SE)**							
	Month 6	−0.18 (0.26)	−0.44 (0.26)	−0.16 (0.25)	−0.27 (−1.00 to 0.7)	0.02 (−0.70 to 0.73)	0.28 (−0.44 to 1.01)
	Month 12	0.35 (0.31)	−0.54 (0.32)	−0.52 (0.30)	−0.88 (−1.76 to −0.002)	−0.87 (−1.73 to −0.01)	0.01 (−0.85 to 0.88)

^a^Denominators vary because of missing data.

^b^Calculated as weight in kilograms divided by height in meters squared.

^c^Confidence intervals that do not contain zero have a *P* value <.05.

### Perceptions of IVR Self-Monitoring

Generally, Shape participants perceived IVR self-monitoring positively. Most (89%, 73/82) agreed that the IVR calls made it easy to self-monitor behavioral goals and 72% (59/82) strongly disagreed that using IVR-based self-monitoring was difficult. A majority (62%, 50/81) reported that the IVR calls were enjoyable and more than half (56%, 46/82) reported that it was easy to fit self-monitoring via IVR into their daily routine. Most (83%, 67/81) reported that IVR self-monitoring made it easy to share information with their Shape coaches; however, only 7% (6/83) said that they answered the IVR calls because they knew the coaches would see their data. Rather, a majority of participants (66%, 55/83) reported that the motivation for answering the IVR calls was to stay on track with their behavioral goals. Most (84%, 68/81) said weekly self-monitoring via IVR was the appropriate frequency and 91% (73/80) reported that the duration of the calls was just right.

## Discussion

### Principal Findings

We found high adherence to weekly IVR self-monitoring calls among low-income black women enrolled in a weight gain prevention intervention. Over the 12-month intervention, nearly three-quarters were adherent to the self-monitoring protocol and more than half of the women completed at least 80% of the 52 IVR calls. Adherence was higher for older, more educated women. Although weight loss was unintended in this trial, we found a positive relation between self-monitoring adherence and weight change; those who completed at least 80% of calls lost almost 2.5 kg more than those with lower adherence. We provided daily paper-based logs as an additional self-monitoring option, but use of the paper-based approach did not enhance IVR adherence or weight loss outcomes. Most participants reported that IVR self-monitoring was easy, helpful, and fit into their daily routine. Compliance with the monthly coaching calls also helped enhance adherence to IVR self-monitoring. We conclude that IVR self-monitoring is effective, produces high adherence rates, and has the potential for greater sustainability in a socioeconomically disadvantaged patient population.

Self-monitoring adherence is one of the strongest predictors of weight outcomes [7]. eHealth approaches can enhance self-monitoring adherence by reducing some of the barriers typically associated with paper-based approaches; however, adherence rates do vary by eHealth modality. Burke and colleagues tested the use of a personal digital assistant (PDA) with or without feedback compared to paper-based self-monitoring within the context of a weight loss intervention and found that less than half of participants remained adherent to self-monitoring using PDAs, and only 30% remained adherent to the paper-based approach at 6 months [37]. By 18 months, rates of adherence decreased below 20% for all groups [9]. Tate and colleagues found that adherence to a Web-based self-monitoring food and exercise diary averaged around 50% by the end of a 12-month intervention [38]. Despite the portability and convenience of text messaging for self-monitoring, adherence rates to text message self-monitoring also average around 50-60% [39,40]. We found that IVR self-monitoring produced higher adherence compared to these other eHealth self-monitoring approaches and remains significantly higher than paper-based methods. Given that high adherence equates to better behavior change outcomes, IVR may be more effective than other eHealth approaches; however, comparative effectiveness studies are needed.

Our high adherence rates may be a result of the type of self-monitoring and frequency of self-monitoring required in addition to the mode through which participants’ monitored. Most weight loss trials ask participants to keep a detailed daily diary of complex aspects of dietary intake and exercise. This requires participants to measure food and perform mathematical functions such as counting calories or grams of fat. This can be difficult to sustain for extended periods of time. Indeed, adherence to these approaches declines over time [21]. Our study, in contrast, asked participants to track weekly a limited number (3) of discrete, simple behavior change goals associated with weight loss (eg, eat 5 or more fruits and vegetables per day, no sugary drinks, 10,000 steps/day) in order to achieve the desired caloric deficit. Self-monitoring of specific behaviors rather than detailed dietary records may be less burdensome and therefore easier to continue over long periods of time. It may also be easier to recall during brief weekly IVR calls. Given our study design, it is not possible determine whether the mode, frequency, or type of self-monitoring was driving the high adherence rates.

This study is among the first to provide detailed evidence for the utility of IVR self-monitoring for weight gain prevention. To our knowledge, only two other studies [22,23] have utilized IVR in a weight loss trial. Estabrooks and colleagues [22] similarly found a 75% adherence rate in their 3-month weight loss trial; although participants were only required to complete 12 calls, while our trial included 52 calls. Additionally, participants in the Estabrooks et al study were predominantly white (69%; 5% black) and more socioeconomically advantaged than Shape participants [22]. Bennett and colleagues [23] also utilized IVR as an intervention tool within a 24-month randomized-controlled effectiveness trial among older, obese, hypertensive patients with similar sociodemographic characteristics to our study, although both males and females were included. Intervention participants could choose to self-monitor their progress on behavior change goals using either IVR or a study website, with the majority of participants (61%) choosing IVR. In terms of adherence, the IVR call completion rates were similar to other eHealth modalities; on average, 57% of IVR calls were completed at 12 months and 48% by 24 months [23]. Our higher adherence rates may be a result of a number of methodological and sociodemographic differences, such as age, comfort with technology, and the fact that our study focused on weight gain prevention while the Bennett et al study focused on achieving weight loss.

IVR has particular promise for socioeconomically disadvantaged populations because it is telephone-based and does not rely on Internet connectivity as is required for other eHealth approaches (eg, Web-based, mobile tracking). These findings are important as these populations, particularly black women, have the highest prevalence of obesity compared to any other group [6], and achieving clinically meaningful weight loss among low-income black women, in particular, has been challenging [41]. Across numerous studies, black women achieve smaller weight losses compared to other groups [42]. It is not clear whether self-monitoring adherence varies by race/ethnicity, as there appear to be no studies in the literature that have examined racial/ethnic disparities in adherence rates. One might suspect that the poorer weight loss outcomes might be, in part, driven by poorer adherence rates. We found that 50% of the sample achieved greater than 80% IVR call completion, which was associated with greater weight loss. Given these high rates of adherence, IVR would appear indicated in future weight control interventions that target these high-risk populations.

### Strengths and Limitations

Our study is one of the first to examine the utility of IVR technology for self-monitoring within a weight management intervention. We examined self-monitoring adherence among a population typically underrepresented in weight control research and for whom obesity treatment is of clinical importance. We had a long follow-up period and maintained high adherence and retention throughout the 52-week intervention. Furthermore, we tested a unique goal-oriented self-monitoring approach for self-monitoring that is less cumbersome compared to more traditional detailed monitoring. This approach may be more effective and sustainable, particularly for high-risk populations. Although we sustained high adherence at 12 months, longer-term follow-up would help determine the true sustainability of an IVR-based approach. Our findings are conservative as we chose to report adherence rates among all eligible intervention participants and not disaggregate participants who experienced technical problems from those who chose to stop intervention activities. Future research would benefit from a more detailed account of the potential causes of low adherence. With the current study design, it is not clear whether IVR self-monitoring is more effective than other eHealth modes. Comparative effective studies are necessary to determine the most effective approach for self-monitoring. Last, this study examined the utility of IVR self-monitoring within the context of a weight maintenance intervention among black women in the primary care setting; thus, we cannot infer whether IVR as the main self-monitoring strategy would be similarly effective within the context of a weight loss intervention in different populations and settings.

### Conclusions

IVR technology is a promising goal-oriented self-monitoring tool within weight control interventions, particularly for high-risk populations. Using this technology produced adherence rates that were higher than other eHealth approaches to self-monitoring. It was also more favorably received than other approaches to self-monitoring. Given the ubiquity of mobile phones, particularly among racial/ethnic minority populations [5,43], IVR can be a useful tool to promote self-monitoring and facilitate intervention delivery.

## Multimedia Appendix 1

Sample IVR call.

## Multimedia Appendix 2

CONSORT-­EHEALTH checklist V1.6.2 [44].

## Abbreviations

ANOVA: analysis of variance
BMI: body mass index
ICC: intraclass correlation coefficient
iOTA: interactive obesity treatment approach
IVR: interactive voice response
MOS-SSS: Medical Outcomes Study-Social Support Survey
PDA: personal digital assistant
PHS: Piedmont Health Services
