# Supplementary material for: Adherence to Self-Monitoring via Interactive Voice Response Technology in an eHealth Intervention Targeting Weight Gain Prevention Among Black Women: Randomized Controlled Trial
Source: J Med Internet Res. 2014 Apr 29;16(4):e114. doi: 10.2196/jmir.2996 (PMC4019773; doi:10.2196/jmir.2996)
Supplement: Supplementary file 2 [file jmir_v16i4e114_app2.pdf]

CONSORT-EHEALTH Checklist V1.6.2 Report

(based on CONSORT-EHEALTH V1.6), available at [http://tinyurl.com/consort-ehealth-v1-6].

2996

Date completed
9/30/2013 12:53:17
by
Dori

Adherence to IVR self-monitoring in an eHealth intervention targeting weight gain prevention among Black women

TITLE

1a-i) Identify the mode of delivery in the title
Adherence to self-monitoring via interactive voice response technology in an eHealth intervention targeting weight gain prevention among Black women
1a-ii) Non-web-based components or important co-interventions in title
"interactive voice response technology"
1a-iii) Primary condition or target group in the title
"among Black women"

ABSTRACT

1b-i) Key features/functionalities/components of the intervention and comparator in the METHODS section of the ABSTRACT
"Intervention participants (n=91) used IVR technology to self-monitor behavior change goals (e.g., no sugary drinks, 10,000 steps per day) via weekly IVR calls."
1b-ii) Level of human involvement in the METHODS section of the ABSTRACT
n/a - although the intervention included human contact, we are not presenting data on human involvement in this manuscript.
1b-iii) Open vs. closed, web-based (self-assessment) vs. face-to-face assessments in the METHODS section of the ABSTRACT
n/a - this information is not included in the abstract. We did, however, report the following:

"Weight data were collected in clinic at baseline, 6, and 12 months. Self-monitoring data was stored in a study database and adherence was operationalized as the percent of weeks with a successful IVR call."
1b-iv) RESULTS section in abstract must contain use data
"Over 12 months, the average IVR completion rate was 71.6% ± 28.1% and 52% (n=47) had an IVR completion rate ≥80%."
1b-v) CONCLUSIONS/DISCUSSION in abstract for negative trials
"Adherence to IVR self-monitoring was high among socioeconomically disadvantaged Black women enrolled in a weight gain prevention intervention."

INTRODUCTION

2a-i) Problem and the type of system/solution
"...evidence indicates that self-monitoring is highly predictive of weight loss success.[7, 8] Despite its effectiveness, adherence to traditional paper-based approaches declines rapidly over time.[7, 9]"

"Despite the growing literature surrounding the use of IVR technologies, limited evidence exists on the use and effectiveness of IVR for weight control."

"We sought to examine the association between IVR self-monitoring and weight change among socioeconomically disadvantaged Black women enrolled in the Shape Program ("Shape")."
2a-ii) Scientific background, rationale: What is known about the (type of) system
"IVR allows participants to interact with a computer system via outbound or inbound telephone calls using the keypad or speech. Use of IVR is ubiquitous in the wider consumer market and, given its widespread familiarity, might be an effective way to collect self-monitoring data within health interventions. Indeed, IVR has been used in a variety of clinical contexts as a means of both delivering intervention content and collecting data.[13-17] IVR may have a number of distinct advantages over the use of other eHealth modalities.[18] It may be particularly useful for low literacy populations; the task of listening to a voice prompt and responding with a simple numerical answer can be far less cognitively and numerically demanding than producing detailed reports of self-monitoring data (e.g., caloric intake or fat intake). IVR calls can also be less time-consuming than other modalities for self-monitoring that might require participants to log onto an online system or conduct an extensive search for required numerical data (e.g., calorie intake). Additionally, IVR systems can be used to provide dynamic and immediate feedback in response to self-monitoring data. Indeed, evidence indicates improvements in health outcomes with the use of IVR for self-monitoring.[17] Despite the growing literature surrounding the use of IVR technologies, limited evidence exists on the use and effectiveness of IVR for weight control."

METHODS

3a) CONSORT: Description of trial design (such as parallel, factorial) including allocation ratio
"In the present analysis, we describe patterns of IVR self-monitoring adherence over time, examine selected predictors of adherence, and explore the association between adherence and weight change."
3b) CONSORT: Important changes to methods after trial commencement (such as eligibility criteria), with reasons
n/a
3b-i) Bug fixes, Downtimes, Content Changes
n/a
4a) CONSORT: Eligibility criteria for participants
"The Shape Program design and methods have been detailed elsewhere.[21, 22] "

"Participants were Black women, aged 25 to 44 years, with a body mass index (BMI) of 25-34.9 kg/m2."
4a-i) Computer / Internet literacy
n/a
4a-ii) Open vs. closed, web-based vs. face-to-face assessments:
"The Shape Program design and methods have been detailed elsewhere.[21, 22] "

"Participants were recruited via mail from five community health centers operated by Piedmont Health (PHS) in central North Carolina using abstracted data on potentially eligible participants."
4a-iii) Information giving during recruitment
"The Shape Program design and methods have been detailed elsewhere.[21, 22]"

"Following eligibility screening, informed consent, and baseline measures,..."

4b) CONSORT: Settings and locations where the data were collected
"in central North Carolina"

"After self-monitoring data was collected and stored in a study database"
4b-i) Report if outcomes were (self-)assessed through online questionnaires
"All measures were administered via online questionnaires."

"At 12 months, participants self-reported via an online questionnaire the average number of days per week they used the paper log, (i.e., 5-7 days per week, 3-4 days per week, 1-2 days per week, or not at all). We also assessed perceptions about IVR self-monitoring at 12 months using an online questionnaire."
4b-ii) Report how institutional affiliations are displayed
n/a
5) CONSORT: Describe the interventions for each group with sufficient details to allow replication, including how and when they were actually administered
5-i) Mention names, credential, affiliations of the developers, sponsors, and owners
n/a
5-ii) Describe the history/development process
"The intervention utilized the interactive obesity treatment approach (iOTA), which has been extensively tested in previous studies.[20, 23]"
5-iii) Revisions and updating
n/a
5-iv) Quality assurance methods
"All IVR call logic was rigorously tested and continuous quality control protocols were performed to ensure fidelity to protocol."
5-v) Ensure replicability by publishing the source code, and/or providing screenshots/screen-capture video, and/or providing flowcharts of the algorithms used
"Figure 2: IVR Call Logic"

"A sample call can be heard in the multimedia appendix."

5-vi) Digital preservation
n/a
5-vii) Access
"The IVR system called participants once a week at a predetermined time."
5-viii) Mode of delivery, features/functionalities/components of the intervention and comparator, and the theoretical framework
"Participants self-monitored these goals throughout the 12-month intervention via weekly IVR phone calls. The IVR calls were on average 2-4 minutes in duration. The IVR system called participants once a week at a predetermined time. If a participant was not reached on the initial attempt, an extensive retry protocol was put into place, with a maximum of 16 attempts over two days."
5-ix) Describe use parameters
"The IVR calls were on average 2-4 minutes in duration. The IVR system called participants once a week at a predetermined time."
5-x) Clarify the level of human involvement
n/a
5-xi) Report any prompts/reminders used
"The IVR system called participants once a week at a predetermined time. If a participant was not reached on the initial attempt, an extensive retry protocol was put into place, with a maximum of 16 attempts over two days."
5-xii) Describe any co-interventions (incl. training/support)
"The Shape intervention included five main components: 1) behavior change goals known to promote weight management; 2) self-monitoring of these goals via weekly IVR phone calls; 3) tailored skills training materials; 4) monthly interpersonal counseling calls with a PHS registered dietitian (“Shape coach”); and 5) a 12-month YMCA membership."
6a) CONSORT: Completely defined pre-specified primary and secondary outcome measures, including how and when they were assessed
"Self-monitoring adherence was calculated as the proportion of intervention participants who successfully completed IVR calls over the number of expected to complete a call by study week"

"We selected several baseline sociodemographic variables and psychosocial constructs that might predict self-monitoring adherence. "

"All measures were administered via online questionnaires."
6a-i) Online questionnaires: describe if they were validated for online use and apply CHERRIES items to describe how the questionnaires were designed/deployed
none reported
6a-ii) Describe whether and how “use” (including intensity of use/dosage) was defined/measured/monitored
"Self-monitoring adherence was calculated as the proportion of intervention participants who successfully completed IVR calls over the number of expected to complete a call by study week. Calls were deemed successful once data on each of the three goals were received."
6a-iii) Describe whether, how, and when qualitative feedback from participants was obtained
n/a
6b) CONSORT: Any changes to trial outcomes after the trial commenced, with reasons
none
7a) CONSORT: How sample size was determined
7a-i) Describe whether and how expected attrition was taken into account when calculating the sample size
reported in main outcomes paper, which is referenced with the statement below:

"The Shape Program design and methods have been detailed elsewhere.[21, 22]"
7b) CONSORT: When applicable, explanation of any interim analyses and stopping guidelines
n/a
8a) CONSORT: Method used to generate the random allocation sequence

reported in main outcomes paper, which is referenced with the statement below:

"The Shape Program design and methods have been detailed elsewhere.[21, 22]"

8b) CONSORT: Type of randomisation; details of any restriction (such as blocking and block size)

reported in main outcomes paper, which is referenced with the statement below:

"The Shape Program design and methods have been detailed elsewhere.[21, 22]"

9) CONSORT: Mechanism used to implement the random allocation sequence (such as sequentially numbered containers), describing any steps taken to conceal the sequence until interventions were assigned

reported in main outcomes paper, which is referenced with the statement below:

"The Shape Program design and methods have been detailed elsewhere.[21, 22]"

10) CONSORT: Who generated the random allocation sequence, who enrolled participants, and who assigned participants to interventions

reported in main outcomes paper, which is referenced with the statement below:

"The Shape Program design and methods have been detailed elsewhere.[21, 22]"

11a) CONSORT: Blinding - If done, who was blinded after assignment to interventions (for example, participants, care providers, those assessing outcomes) and how

11a-i) Specify who was blinded, and who wasn't

reported in main outcomes paper, which is referenced with the statement below:

"The Shape Program design and methods have been detailed elsewhere.[21, 22]"

11a-ii) Discuss e.g., whether participants knew which intervention was the "intervention of interest" and which one was the "comparator"

n/a - comparing to usual care

11b) CONSORT: If relevant, description of the similarity of interventions

n/a - comparing to usual care

12a) CONSORT: Statistical methods used to compare groups for primary and secondary outcomes

"All analyses were conducted within the intervention group only (n=91). Descriptive statistics were conducted to characterize the sample and examine average IVR completion rate over the 12-month period. IVR adherence was dichotomized using a median split (80% or more) to examine differences in outcomes among high completers compared to those below the median. Adherence was also analyzed as tertiles of successful weekly IVR calls. We conducted bivariate analyses using t-tests and chi-square to examine potential predictors of average IVR completion rate and categories of IVR completion. Pearson correlations examined the relationship between weight and BMI change and IVR call completion rate. ANOVA analyzed differences in weight change and BMI change among high and low IVR completers, tertiles of IVR completion, and categories of IVR and paper log completion."

12a-i) Imputation techniques to deal with attrition / missing values

none reported in this paper.

12b) CONSORT: Methods for additional analyses, such as subgroup analyses and adjusted analyses

"All analyses were conducted within the intervention group only (n=91). Descriptive statistics were conducted to characterize the sample and examine average IVR completion rate over the 12-month period. IVR adherence was dichotomized using a median split (80% or more) to examine differences in outcomes among high completers compared to those below the median. Adherence was also analyzed as tertiles of successful weekly IVR calls. We conducted bivariate analyses using t-tests and chi-square to examine potential predictors of average IVR completion rate and categories of IVR completion. Pearson correlations examined the relationship between weight and BMI change and IVR call completion rate. ANOVA analyzed differences in weight change and BMI change among high and low IVR completers, tertiles of IVR completion, and categories of IVR and paper log completion. "

RESULTS

13a) CONSORT: For each group, the numbers of participants who were randomly assigned, received intended treatment, and were analysed for the primary outcome

"... we randomized participants (n=194) to either the Shape intervention or usual care arm."

"All analyses were conducted within the intervention group only (n=91)."

13b) CONSORT: For each group, losses and exclusions after randomisation, together with reasons

Listed in Figure 1: Participant Enrollment and Retention (CONSORT)

13b-i) Attrition diagram

Listed in Figure 1: Participant Enrollment and Retention (CONSORT)

14a) CONSORT: Dates defining the periods of recruitment and follow-up

"Started in December 2009, participants were recruited..."

"Final assessments were completed in October 2012."

14a-i) Indicate if critical "secular events" fell into the study period

n/a

14b) CONSORT: Why the trial ended or was stopped (early)

n/a

15) CONSORT: A table showing baseline demographic and clinical characteristics for each group

We did not include a table, as this is available in the main outcomes paper, however, we did provide an overview of baseline characteristics in the text.

"Baseline characteristics and main outcomes have been reported in detail elsewhere.[21, 22] Briefly, participants reported at baseline a mean age of 35.4 ± 5.5 years and a mean BMI of 30.2 ± 2.5. Most (71%) were currently employed with an annual income < $30,000/year (74%). The majority (80%) had less than a college degree."

15-i) Report demographics associated with digital divide issues

All participants were Black females. We reported income and eductation.

"Most (71%) were currently employed with an annual income < $30,000/year (74%). The majority (80%) had less than a college degree."

16a) CONSORT: For each group, number of participants (denominator) included in each analysis and whether the analysis was by original assigned groups

16-i) Report multiple "denominators" and provide definitions

"All analyses were conducted within the intervention group only (n=91)."

16-ii) Primary analysis should be intent-to-treat
"Among all intervention participants (n=91), the average IVR completion rate over 12 months was 71.6% ± 28.1% with a range from 52% to 96%. Similar results are seen among all attempted participants; this rate excludes participants at each study week that may have requested to suspend or stop intervention activities and/or experienced technical problems with the IVR system (n=82 at week 52)."
17a) CONSORT: For each primary and secondary outcome, results for each group, and the estimated effect size and its precision (such as 95% confidence interval)
"the average IVR completion rate over 12 months was 71.6% ± 28.1% with a range from 52% to 96%."

"At 12 months, participants with an IVR completion rate of at least 80% had greater weight loss outcomes compared to those with an IVR completion rate of less than 80% [mean difference (95% CI): -2.45 kg (-4.37, -0.54); p=.01]. Similar findings were seen for 12-month change in BMI [mean difference (95% CI): -0.94 kg/m2 (-1.64, -0.24); p=.009]."
17a-i) Presentation of process outcomes such as metrics of use and intensity of use
"Fifty-two percent of intervention participants (n=47) had an IVR completion rate of 80% or more and two-thirds (66%) completed at least 60% of IVR calls. Throughout the 12-month period, 39% of IVR calls were completed on the first attempt and 78% of calls were completed by the third attempt. About half of participants (49.4%) self-reported using the paper tracking log at least 5 days each week in order to relay behavioral goal attainment to the weekly IVR calls. "
17b) CONSORT: For binary outcomes, presentation of both absolute and relative effect sizes is recommended
n/a
18) CONSORT: Results of any other analyses performed, including subgroup analyses and adjusted analyses, distinguishing pre-specified from exploratory
"Similar results are seen among all attempted participants; this rate excludes participants at each study week that may have requested to suspend or stop intervention activities and/or experienced technical problems with the IVR system (n=82 at week 52). "
18-i) Subgroup analysis of comparing only users
"Similar results are seen among all attempted participants; this rate excludes participants at each study week that may have requested to suspend or stop intervention activities and/or experienced technical problems with the IVR system (n=82 at week 52). "
19) CONSORT: All important harms or unintended effects in each group
n/a
19-i) Include privacy breaches, technical problems
"Similar results are seen among all attempted participants; this rate excludes participants at each study week that may have requested to suspend or stop intervention activities and/or experienced technical problems with the IVR system (n=82 at week 52)."

"Our findings are conservative as we chose to report adherence rates among all eligible intervention participants and not disaggregate participants who experienced technical problems from those who chose to stop intervention activities."
19-ii) Include qualitative feedback from participants or observations from staff/researchers
no qualitative outcomes reported.

DISCUSSION

20) CONSORT: Trial limitations, addressing sources of potential bias, imprecision, multiplicity of analyses
20-i) Typical limitations in ehealth trials
"Although we sustained high adherence at 12 months, longer-term follow-up would help determine the true sustainability of an IVR-based approach. Our findings are conservative as we chose to report adherence rates among all eligible intervention participants and not disaggregate participants who experienced technical problems from those who chose to stop intervention activities. Future research would benefit from a more detailed account of the potential causes of low adherence. With the current study design, it is not clear whether IVR self-monitoring is more effective than other eHealth modes. Comparative effective studies are necessary to determine the most effective approach for self-monitoring."
21) CONSORT: Generalisability (external validity, applicability) of the trial findings
21-i) Generalizability to other populations
"Lastly, this study examined the utility of IVR self-monitoring within the context of a weight maintenance intervention among Black women in the primary care setting; thus, we cannot infer whether IVR as the main self-monitoring strategy would be similarly effective within the context of a weight loss intervention in different populations and settings."
21-ii) Discuss if there were elements in the RCT that would be different in a routine application setting
n/a
22) CONSORT: Interpretation consistent with results, balancing benefits and harms, and considering other relevant evidence
22-i) Restate study questions and summarize the answers suggested by the data, starting with primary outcomes and process outcomes (use)
"We found high adherence to weekly IVR self-monitoring calls among low-income, Black women enrolled in a weight gain prevention intervention."

"Thus, IVR self-monitoring is effective, produces high adherence rates, and has the potential for greater sustainability in a socioeconomically disadvantaged patient population. "
22-ii) Highlight unanswered new questions, suggest future research
"Given that high adherence equates to better behavior change outcomes, IVR may be more effective than other eHealth approaches; however, comparative effectiveness studies are needed."

"Although we sustained high adherence at 12 months, longer-term follow-up would help determine the true sustainability of an IVR-based approach."

"Future research would benefit from a more detailed account of the potential causes of low adherence."

"thus, we cannot infer whether IVR as the main self-monitoring strategy would be similarly effective within the context of a weight loss intervention that also included support from coaches."

Other information

23) CONSORT: Registration number and name of trial registry
"Trial Registration: clinicaltrials.gov NCT00938535 (http://www.clinicaltrials.gov/ct2/show/NCT00938535)"
24) CONSORT: Where the full trial protocol can be accessed, if available

"The Shape Program design and methods have been detailed elsewhere.[21, 22]"

"21. Foley P, Levine E, Askew S, Puleo E, Whiteley J, Batch B, et al. Weight gain prevention among black women in the rural community health center setting: the Shape Program. BMC Public Health. 2012;12:305.

22.Bennett GG, Foley P, Levine E, Whiteley J, Askew S, Steinberg DM, et al. Behavioral Treatment for Weight Gain Prevention Among Black Women in Primary Care Practice: A Randomized Clinical Trial. JAMA Intern Med. 2013."

25) CONSORT: Sources of funding and other support (such as supply of drugs), role of funders

"Funding and Support: This trial is funded by grant R01DK078798 from the National Institute for Diabetes and Digestive and Kidney Diseases. Dr. Bennett was supported by K22CA126992. The National Institute for Diabetes and Digestive and Kidney Diseases had no role in the design and conduct of the study; the collection, management, analysis, and interpretation of the data; or preparation, review, or approval of the manuscript. "

X26-i) Comment on ethics committee approval

"The relevant university and health system review boards approved all study procedures."

x26-ii) Outline informed consent procedures

"Following eligibility screening, informed consent, and baseline measures, we randomized participants (n=194) to either the Shape intervention or usual care arm."

X26-iii) Safety and security procedures

n/a

X27-i) State the relation of the study team towards the system being evaluated

"Conflicts of Interest: The authors declare no conflicts of interest. "
